# Divergence of RNA polymerase α subunits in angiosperm plastid genomes is mediated by genomic rearrangement

**DOI:** 10.1038/srep24595

**Published:** 2016-04-18

**Authors:** J. Chris Blazier, Tracey A. Ruhlman, Mao-Lun Weng, Sumaiyah K. Rehman, Jamal S. M. Sabir, Robert K. Jansen

**Affiliations:** 1 Department of Integrative Biology, University of Texas, Austin, TX 78712, USA; 2 Biotechnology Research Group, Department of Biological Science, Faculty of Sciences, King Abdulaziz University, Jeddah 21589, Saudi Arabia

## Abstract

Genes for the plastid-encoded RNA polymerase (PEP) persist in the plastid genomes of all photosynthetic angiosperms. However, three unrelated lineages (Annonaceae, Passifloraceae and Geraniaceae) have been identified with unusually divergent open reading frames (ORFs) in the conserved region of *rpoA*, the gene encoding the PEP α subunit. We used sequence-based approaches to evaluate whether these genes retain function. Both gene sequences and complete plastid genome sequences were assembled and analyzed from each of the three angiosperm families. Multiple lines of evidence indicated that the *rpoA* sequences are likely functional despite retaining as low as 30% nucleotide sequence identity with *rpoA* genes from outgroups in the same angiosperm order. The ratio of non-synonymous to synonymous substitutions indicated that these genes are under purifying selection, and bioinformatic prediction of conserved domains indicated that functional domains are preserved. One of the lineages (*Pelargonium*, Geraniaceae) contains species with multiple *rpoA*-like ORFs that show evidence of ongoing inter-paralog gene conversion. The plastid genomes containing these divergent *rpoA* genes have experienced extensive structural rearrangement, including large expansions of the inverted repeat. We propose that illegitimate recombination, not positive selection, has driven the divergence of *rpoA*.

Before inexpensive DNA sequencing, the plastid genomes (plastomes) of flowering plants (angiosperms) were surveyed for gene content using Southern hybridization^1^,^2^,^3^. These surveys revealed remarkably conserved gene order and content across almost all angiosperms, yet also discovered a few isolated lineages with highly divergent, rearranged plastomes lacking genes and introns. The subsequent publication of more than 800 complete plastomes has confirmed most of these early results. Plastomes typically contain 79 protein-coding genes, 30 tRNA and 4 rRNA genes^4^. Plastid encoded genes are often categorized as either photosynthesis related or housekeeping, and the latter are generally found to have been lost from plastomes and either functionally replaced or transferred to the nucleus^5^,^6^,^7^. Among the housekeeping genes encoded by the plastome are the four subunits of the eubacterial-like RNA polymerase (PEP) that is responsible for most photosynthetic gene expression^8^.

The genes encoding the three largest PEP subunits, β, β′ and β″, are cotranscribed from *rpoB*, *rpoC1* and *rpoC2,* respectively. The α subunit is encoded by *rpoA*, the last gene in the conserved *rpl23* transcriptional unit consisting mostly of ribosomal protein genes^8^. The three large subunit genes have only been found missing from a few parasitic and mycoheterotrophic plant plastomes^9^,^10^. In these cases it appears that the single-subunit nuclear-encoded RNA polymerase (NEP) has taken over transcription of the residual plastome, which no longer encodes a functional photosynthetic apparatus^7^. All deletions of individual PEP subunits from *Nicotiana tabacum* (tobacco) produced photosynthetically defective transformants, demonstrating that each of the four subunits is an essential gene^11^. PEP and NEP transcribe many of the same genes using distinct promoters; species lacking PEP have also lost PEP-specific promoters and nuclear-encoded σ factors^12^,^13^.

Southern hybridization surveys identified three unrelated lineages, *Pelargonium* (Geraniaceae), *Annona* (Annonaceae) and *Passiflora* (Passifloraceae), which appeared to lack the plastid *rpoA* gene^1^,^14^. Subsequent work^6^ identified highly divergent *rpoA* sequences encoded in the plastomes of *P. x hortorum* and *Passiflora biflora*, however no further data are available for *Annona*.

The *P. x hortorum* plastome is the largest and most complex angiosperm plastome yet discovered and houses three distinct, divergent *rpoA*-like ORFs^15^. No other plastome is known to harbor multiple paralogs of this gene, and it is difficult to judge which, if any, of these divergent genes are functional. Moreover, it is unclear whether they have diverged due to positive or relaxed selection or by some unusual, locus-specific neutral process.

Determining the functionality of *rpoA* poses several difficulties. Due to its location at the end of a conserved transcriptional unit, mRNA expression data are uninformative, as it has been shown that the entire plastome can be transcribed via read-through^16^. There is no published nuclear genome data for *Pelargonium, Passiflora or Annona*. It is possible that *rpoA* has been transferred to the nucleus and that the divergence of the gene reflects relaxed selection on the plastid copy in the wake of its functional replacement by a nuclear paralog. Although the transfer of *rpoA* has not been demonstrated in angiosperms, it was detected in the moss *Physcomitrella patens*^17^, and it was inferred that *rpoA* has been transferred to the nucleus twice in the bryophytes^18^. Following functional transfer to the nucleus, the original plastome gene copy may degrade slowly, making it difficult to judge the functionality of an ORF if the gene has been transferred relatively recently^19^.

Due to the intractability of reverse genetics in most plastomes, we have adopted a sequence-based approach to address whether *Pelargonium, Passiflora and Annona* plastomes still encode a functional PEP α subunit. We conducted substitution rate analyses to explore selective forces acting on the *rpoA* sequences in these plastomes. The results of our *in silico* analyses suggest that these *rpoA*-like sequences are functional genes, some of which have been evolving in manners unlike that of other plastid genes due to illegitimate recombination. Furthermore, illegitimate recombination is also evident in the large changes in the inverted repeat (IR) boundaries in all three lineages.

## Results

### Plastome sequence of *Annona cherimola*

The plastome of *Annona cherimola* is 201,723 bp with a 69,771 bp large single copy (LSC) region, a 64,493 bp IR and a small single copy (SSC) region of only 2,966 bp (Fig. S1). The IR has greatly expanded at both the IR_B_/SSC and IR_B_/LSC boundaries. Expansions at the IR_B_/LSC boundary duplicated 24 genes, from *rps19* through most of *psbA*. The IR_B_/SSC expansion included 11 genes from *ycf1* through *trnL*-UAG. This resulted in a very small SSC containing a single complete gene (*rpl32*) and a nearly complete copy of *ndhF*. The *Annona* plastome comprises 165 genes: 113 unique genes and 52 duplicated genes in the expanded IR. Gene order is highly conserved compared to the ancestral plastid genome organization for angiosperms^7^ with a single inversion involving six genes (*ycf3* - *atpE*) in the LSC (Fig. S1). Gene content is also highly conserved with no apparent gene loss, however, *rpoA* is highly divergent with a nucleotide sequence identity of 57% compared to *Chloranthus*, which is sister to the magnoliid clade (Table 1).

### High levels of *rpoA* sequence divergence in three unrelated angiosperm lineages

Comparison of both nucleotide and amino acid sequence divergence of *rpoA* for members of the three unrelated lineages of angiosperms, Annonaceae, *Passiflora* (Passifloraceae) and *Pelargonium* (Geraniaceae) were performed (Tables 1, 2). For Annonaceae the three genera examined (*Annona*, *Asimina* and *Cananga*) have nucleotide and amino acid sequence identities ranging from 56–75% and 39–64%, respectively, in comparison to *Chloranthus* (Table 1). This is in contrast to the 86–92% and 80–90% nucleotide and amino acid sequence identities, respectively, for the five other magnoliids examined. Sequence identities within *Passiflora* were variable for one species, *P. biflora*, which showed high levels of *rpoA* divergence, 54% and 37% nucleotide and amino acid sequence identities, respectively (Table 1). Levels of sequence identity of the other three species of *Passiflora* and eight species from other families of rosids were substantially higher (81–94% and 71–91%, respectively). Within *Pelargonium*, levels of sequence identity of *rpoA* were among the lowest, with nucleotide and amino acid identities ranging from 30–49% and 15–34%, respectively (Table 2). The levels of sequence divergence are much lower in related rosids (78–92% nucleotide and 65–86% amino acid identity), including four other genera of Geraniales, one of which is a member of the Geraniaceae (i.e., *Hypseocharis*).

### Detection of plastid *rpoA* transcripts by RT-PCR

Transcripts were confirmed for the two longer *rpoA*-like ORFs of *P. x* *hortorum*, ORF578 and ORF597 (Fig. S2); there are at least dicistronic transcripts for both of these ORFs. The result does not preclude ORF transcripts being present as monocistrons or as polycistrons, including genes further upstream.

### Conservation of PEP promoters and sigma factors

A database comprising contigs from the published^20^ high-coverage nuclear transcriptome assembly of *P. x hortorum* was queried with *rpoA* nucleotide and amino acid sequences from *A. thaliana*. No nuclear-encoded *rpoA* paralog transcript was detected in either the nucleotide or the translated database by BLAST search. Other nuclear-encoded components of the PEP holoenzyme, e.g. sigma factors, were found using the same BLAST parameters and were recently reported in Zhang *et al.*^21^.

**Figure 1:**
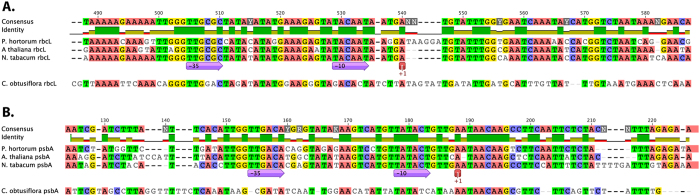
Alignment of PEP promoter regions. (**A**) Alignment of promoter region for *rbcL* in three species with functional PEP (*Nicotiana tabacum*, *Arabidopsis thaliana*, *Pelargonium x hortorum*) and one lacking PEP (*Cuscuta obtusiflora*). (**B**) Alignment of promoter region for *psbA* in three species with functional PEP (*N. tabacum*, *A. thaliana*, *P. x hortorum*) and one lacking PEP (*C. obtusiflora*). The conserved −10 and −35 elements are indicated by block arrows and the transcription start site is indicated by a red box (+1).

*In silico* examination of PEP promoters upstream of the *rbcL* and *psbA* coding regions revealed that *P. x hortorum* sequences closely resembled those of *A. thaliana* and *N. tabacum*. The -35 and -10 elements, as well as the transcription start sites, were 100% identical across all three species, unlike in *Cuscuta obtusiflora*, a parasitic plant lacking PEP (Fig. 1A,B).

### Analysis of signals of selection

**Figure 2:**
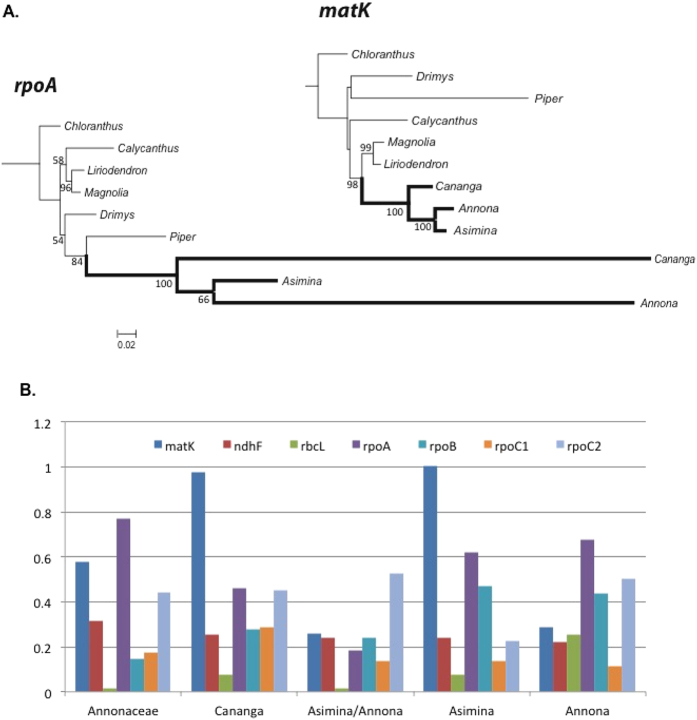
Representative maximum likelihood trees and *dN*/*dS* values for the nine taxa of Annonaceae. (**A**) Likelihood scores for the *matK* and *rpoA* trees were −5638.2661 lnL and −5506.0125 lnL, respectively. Branches in bold are members of Annonaceae. Bootstrap values greater than 50 are shown at the nodes. Scale bar indicates non-synonymous substitutions per codon. (**B**) Histogram of *dN*/*dS* values for seven genes for the Annonaceae. For each gene, *dN*/*dS* values (y axis) are given for all branches of interest: the branch leading to the family, the internal branch to *Annona*/*Asimina*, and the terminal branches to *Annona*, *Asimina*, and *Cananga*. Only one ratio was marginally >1, the terminal branch to *Asimina* for *matK* (*dN*/*dS* = 1.0069).

**Figure 3:**
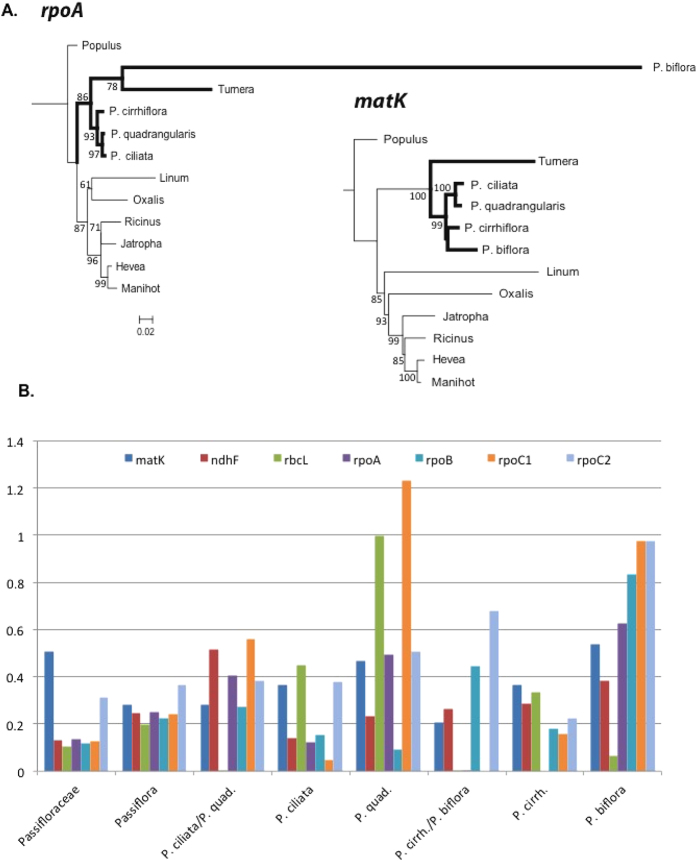
Representative maximum likelihood trees and *dN*/*dS* values for 12 taxa of Passifloraceae. (**A**) Likelihood scores for the *matK* and *rpoA* trees were lnL −7243.3426 and −4810.9045 lnL, respectively. Branches in bold are members of Passifloraceae. Bootstrap values greater than 50 are shown at the nodes. Scale bar indicates non-synonymous substitutions per codon. (**B**) Histogram of *dN*/*dS* ratios for seven genes for the Passifloraceae. For each gene, *dN*/*dS* values (y axis) are given for all branches of interest: the branch leading to the family as well as all internal and terminal branches. The primary branch of interest is the terminal branch to *P. biflora*, the only species with a divergent *rpoA* gene. The terminal branch to *P. quadrangularis* for *rpoC1* has a *dN*/*dS* value >1, but this is likely an artifact, as the branch length is extremely short. The lack of a bar for *rbcL* is due to a *dS* value of 0.

**Figure 4:**
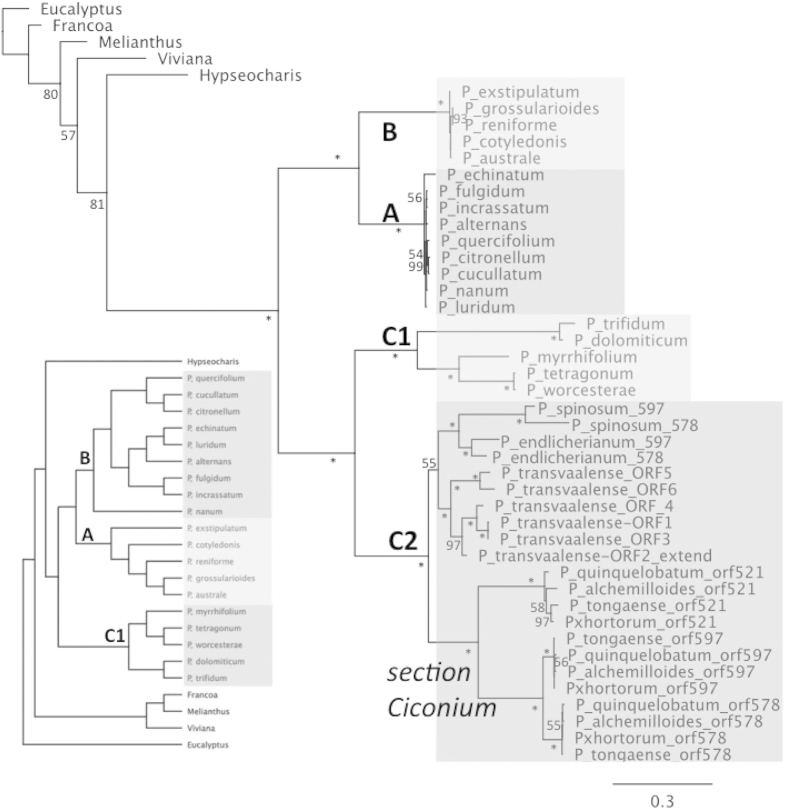
Maximum likelihood tree generated for all 46 *rpoA* ORFs from 26 *Pelargonium* species with likelihood score −21428.281249 lnL. Species in clade C2 contain two (*P. spinosum and P. endlicherianum*), three (four species from section *Ciconium*) or six (*P. transvaalense*) *rpoA* paralogs. Bootstrap values greater than 50 are shown at the nodes; values of 100 are indicated by asterisks. Scale bar indicates non-synonymous substitutions per codon. The constraint tree (inset) does not contain clade C2 taxa as these species all contain multiple *rpoA* sequences.

The *dN*/*dS* ratio was calculated for the three different lineages of angiosperms. Seven plastid genes (*rpoA*, *rpoB*, *rpoC1*, *rpoC2*, *ndhF*, *matK* and *rbcL*) were analyzed in PAML for three datasets to compare the *dN*/*dS* ratio of *rpoA* to the other *rpo* genes as well as to other non-*rpo* plastid genes. These same seven genes were used to generate constraint trees for each dataset. Constraint tree topologies were identical to the *matK* trees for Annonaceae (Fig. 2A) and Passifloraceae (Fig. 3A). The seven gene constraint tree for Geraniaceae is shown as an inset in Fig. 4.

### Annonaceae

Maximum likelihood trees for *matK* and *rpoA* (Fig. 2A) were generated from the Annonaceae dataset, which comprised eight magnoliids including three genera in the Annonaceae, and *Chloranthus* of the Chloranthales (Table 3). The *matK* tree had the same topology as most other individual plastid genes (not shown) where the branch leading to *Piper* was long but branches within Annonaceae relatively short. However, in the *rpoA* tree branch lengths within Annonaceae were sufficiently long to produce an incorrect topology through long-branch attraction to *Piper*. The five branches of interest are highlighted in Fig. 2B. The terminal branch leading to *Asimina* for *matK* had the only *dN*/*dS* value >1 (1.0069). All *rpo* genes showed *dN*/*dS* values consistent with purifying selection in Annonaceae.

### Passiflora

Maximum likelihood trees for *matK* and *rpoA* were constructed from the *Passiflora* dataset, consisting of 12 taxa from the Malpighiales, including four *Passiflora* species (Fig. 3A, Table 3). The *matK* tree has the same topology as most other individual plastid genes (not shown), with a long branch leading to *Turnera* (Passifloraceae) but relatively short branches within *Passiflora*. In the *rpoA* tree, however, the long terminal branch leading to *P. biflora* resulted in long-branch attraction to *Turnera*. For *dN/dS* ratios, the branches of interest are highlighted in Fig. 3B. The principal branch of interest was the terminal branch leading to *P. biflora*, the only species with a divergent *rpoA*. The only gene for which a branch had a *dN/dS* value >1 (1.2312) was *rpoC1*, on the terminal branch leading to *P. quadrangularis*.

### Pelargonium

The *Pelargonium* dataset consisted of 26 species representing all major clades (Table 3). *Pelargonium rpoA* genes showed a complex pattern of divergence by clade that confounded the analysis of evolutionary rates. A maximum likelihood tree of all *rpoA* genes/ORFs from the *Pelargonium* dataset was generated (Fig. 4). To overcome the potential for error due to the difficulties in aligning *rpoA* sequences across clades and with outgroups four different alignment algorithms were utilized in *Pelargonium* rate comparisons (Table S2).

The *rpoA* genes in clades A and B were somewhat divergent between the two clades, sharing only 66–71% nucleotide sequence identity, but showed high identity within each clade. The five *rpoA* genes representing clade B shared 94% sequence identity. However, this percentage was lowered by indels associated with tandem repeats at the 3′ end of the gene immediately preceding the predicted stop codon (Fig. S3). When this repeat-rich region was excluded from the alignment the remaining sequences share over 98% identity. In fact, four of the five genes were 100% identical when the 3′ end was excluded, and the fifth, *P. exstipulatum*, differed by only two nucleotides, both of which were nested in tandem repeats and caused non-synonymous substitutions.

Nine *rpoA* genes representing clade A shared 92% identical sites, or 95% identical sites if the 3′ end was excluded. Similar to clade B, different numbers of tandem repeats towards the 3′ end caused length differences in clade A *rpoA* (Fig. S4). Although indels associated with tandem repeats underlie the length differences between *rpoA* genes of clades A and B, the repeats were nonhomologous sequences. In clade B there were two different tandem repeat units that underlie the length differences: a 6 bp motif of GCGAGG was present in all the ORFs, ranging from two repeat units in *P. australe* to eight in the same region of *P. grossularioides*. In *P. cotyledonis*, two copies of this 6 bp tandem repeat were nested inside a unique 39 bp repeat, which expanded to four tandem copies, the last base pair of which was the first base pair of the predicted TAA stop codon (Fig. S3). The 6 bp repeat from clade B was not found in any clade A *rpoA* sequence, instead, a 9 bp repeat unit, present as both tandem and dispersed repeats at the 3′ end of the gene in all clade A species, appeared to have caused a deletion of 30 bp between two direct, dispersed 9 bp repeat units in *P. echinatum* and *P. fulgidum*. These two taxa are not sister species, thus it appeared that this deletion occurred twice independently in clade A.

The C1 and C2 clades were highly divergent both within and between clades, and the C2 clade contained species with multiple (2, 3 or 6) *rpoA*-like ORFs (Fig. 4). For clade C2 species it was not clear which of the paralogous ORFs might be functional. ORFs from clade C2 were excluded from *dN*/*dS* analysis (see Gene Conversion below).

Clade C1 was represented by five species whose ORFs fell into two groups of more closely related sequences. *Pelargonium dolomiticum* and *P. trifidum* shared 96% nucleotide sequence identity. *Pelargonium tetragonum* and *P. worcesterae* had 99% identity and were identical in length at 912 bp; *P. myrrhifolium* was more closely related to this second pair but shared only 61% identity with *P. tetragonum*. Between the groups, *P. dolomiticum* and *P. tetragonum* had only 64% identity.

**Figure 5:**
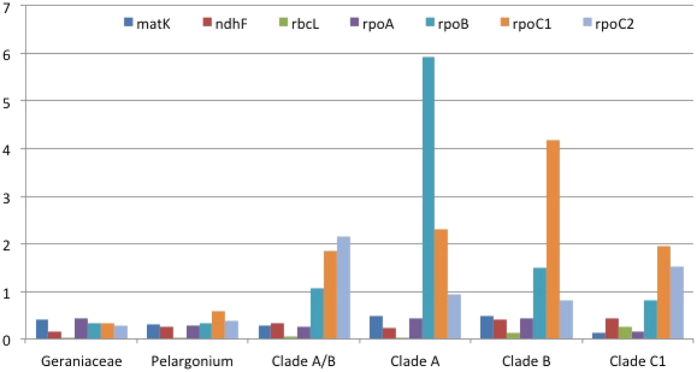
Histogram of *dN*/*dS* ratios for seven genes for Geraniaceae. In addition to MAFFT results presented here, three other alignment algorithms were used (See Table S2). For each gene, *dN*/*dS* values are given for all branches of interest, the branch leading to the family (Geraniaceae), to *Pelargonium*, to the branch to clades A/B, to clade A, to clade B, and to clade C1.

The branches of interest for the *Pelargonium* rates analyses were different from those in the previous two data sets: the terminal branches were excluded as intra-clade divergence among species was extremely low due to dense taxon sampling in this dataset. Low sequence divergence between closely related taxa caused error values to be returned in the calculation of *dN*/*dS* where either or both of the parameters were calculated to be zero or close to zero (not shown). Therefore the branches of interest were chosen as those where the greatest divergence in *rpoA* has occurred and are highlighted in Fig. 5.

Rates analyses of *matK*, *ndhF* and *rbcL* for *Pelargonium* detected low *dN*/*dS* values consistent with purifying selection across all alignments for all branches of interest (Fig. 5; Table S2). For the *rpo* genes, a pattern emerged that was consistent across all alignment methods used: *dN*/*dS* values for *rpoA* were uniformly low (<1), consistent with purifying selection on all branches of interest (Fig. 5; Table S2). However, *dN*/*dS* values for the other *rpo* genes were elevated along several branches of interest (Fig. 5; Table S2). On the branch leading to clades A and B, *rpoB*, *rpoC1* and *rpoC2* all showed *dN*/*dS* values >1. The same was seen for the branches leading to each clade (A and B) except for *rpoC2* on the clade A branch, where *dN*/*dS* values were near or >1 depending on the alignment method used. On the branch leading to the C1 clade, *rpoC1* and *rpoC2* but not *rpoB* showed *dN*/*dS* values >1.

### Detection of conserved domains

For each of the three datasets, *rpoA* genes from the outgroup taxa were queried against the Conserved Domain Database (CDD) for detection of functional domains that lie in the N-terminal region of the α-subunit. In each case the three functional domains, involved in the interaction of the α-subunit with itself and the β and β′ subuints, were predicted as present (Tables 1 and 2). Having verified the predictive capability of the CDD in these conserved plastid genes, all the other *rpoA* genes were queried against the database to predict the presence of the three interaction domains.

In Annonaceae, all *rpoA* ORFs were predicted to encode all three interaction domains including those from *Annona*, *Asimina* and *Cananga*, despite their substantial sequence divergence from the outgroup *Chloranthus* (Table 1). Likewise, in *Passiflora*, all *rpoA* ORFs were predicted to encode the three conserved domains (Table 1). In *Passiflora* the divergence was restricted to a single species surveyed, *P. biflora* (Fig. 3).

In *Pelargonium*, all ORFs were predicted to encode the N-terminal region of the α-subunit as well as the homodimer interface. However, the conservation of functional domains showed a more complex pattern that differed by clade (Table 2). Clade B was the simplest as all five *rpoA* sequences were predicted to contain all three functional domains despite retaining just 44%–49% sequence identity with outgroup *Eucalyptus*.

In *Pelargonium* clade A all nine *rpoA* genes were predicted to encode the N-terminus containing the homodimer interface (Table 2), but the CDD search did not predict the other functional domains for two of the four species in clade A1 (*P. citronellum* and *P. cucullatum*). All five species from clade A2 were predicted to contain all three functional domains. Divergence from *Eucalyptus* in clade A is similar to that in clade B, ranging from 45%–46% sequence identity.

The *Pelargonium* C clade contained the most divergent and puzzling *rpoA*-like ORFs with respect to the prediction of conserved functional domains (Table 2). All five taxa representing clade C1 were predicted to encode the homodimer interface, which spans the beginning and end of the α-subunit N-terminus (Fig. S5), but only *P. tetragonum* and *P. worcesterae* were predicted to contain the other two functional domains (Fig. S5). These two species had the highest sequence identity to the outgroup and at 912 bp were closest in length to *rpoA* in most angiosperms (versus 1014 bp in *Eucalyptus*), whereas the other three C1 taxa had shorter genes of 708 bp–750 bp.

Likewise, CDD analyses identified the α-subunit N-terminal region and homodimer domain in all clade C2 taxa *rpoA*-like ORFs. Using high-coverage Illumina sequence data we found two sequencing errors in the *rpoA*-like ORFs of the published *P. x hortorum* plastome annotation^15^. Both errors were single base pairs missing from ORFs, leading to a premature stop codon (ORF578) and to the division of one long ORF into two shorter ORFs (ORF521, formerly ORF221 and ORF332). The re-annotation of these ORFs was confirmed by comparison with those from the three closely related taxa in section *Ciconium*. After correction the plastomes each contained three long *rpoA*-like ORFs of similar length (1566 bp, 1737 bp, and 1794 bp in *P. x hortorum;* Table 4, Fig. S6). These ORF names were used for the homologous ORFs in the other clade C2 species, even though some differ slightly in length; homology was inferred from synteny.

In the two species containing two *rpoA*-like ORFs, *P. endlicherianum* and *P spinosum*, all ORFs were predicted to encode the homodimer interface, yet neither contained the other two functional domains (Table 2). *Pelargonium transvaalense* contained six *rpoA*-like ORFs predicted to encode the N-terminal domain of the α-subunit and the homodimer interface, however only ORF597–2 contained the other two functional domains. In the four section *Ciconium* taxa, at least one of the ORFs in each species was predicted to encode all three functional domains. One homolog, ORF578, was predicted to encode all domains in all four taxa. Although the length of the other two ORFs varied between species, ORF578 was identical in length at 1737 bp in all four taxa and also displayed the highest percentage (99%) of identical sites across the four species.

### Detection of gene conversion among *rpoA* paralogs

The likelihood tree generated from clade C2 *rpoA*-like ORFs showed a pattern suggesting that gene conversion was an important phenomenon underlying the evolution of these unusual ORFs (Fig. 4). First, ORFs from the two taxa containing only two ORFs grouped together by species and not by ORF, suggesting that these ORFs have not been evolving independently since their duplication in the ancestor of C2 taxa. For example, the two ORFs in *P. endlicherianum* shared only 63–69% sequence identity with those from *P. spinosum*, whereas the ORFs in each species shared 86% and 72% identity with its paralog, respectively. The six ORFs in *P. transvaalense* grouped together as well, despite their apparent common ancestry with the ORFs in section *Ciconium*. For the four section *Ciconium* taxa (Fig. 4), the ORFs grouped by ORF in the likelihood tree rather than by species, despite showing evidence of gene conversion among ORFs, likely reflecting the relatively recent divergence of these taxa.

ORGCONV^22^ found evidence of recombination among ORFs in all four species of section *Ciconium* (Table 5), predicting that gene conversion took place in all species in a region from approximately the 120^th^ to 720^th^ (600 bp) position in alignment of the three ORFs. This was the region predicted by the CDD to encode the N-terminus of the α-subunit containing the functional domains. Visual inspection of alignments for mutations potentially resulting from gene conversion was conducted. A parsimony criterion was used: substitutions common to multiple ORFs within a species but not among homologous ORFs across species were scored as putative gene conversion events (Table 6). Both ORGCONV and manual assessment indicated that gene conversion occurred among paralogs in all four section *Ciconium* species.

## Discussion

Of the PEP subunits, α is the least conserved^23^, so its degree of divergence may not be useful in determining functionality. Likelihood-based calculation of *dN*/*dS* ratios to detect selection may be inappropriate for some of these ORFs, as some appear to be evolving in ways not anticipated by standard evolutionary models. For example, gene conversion, which is known to occur between paralogs, can produce spurious signals of selection under likelihood-based models^24^. Furthermore, alignment error could lead to spurious signals of selection^25^, as some of the divergent *rpoA*-like ORFs share less than 40% amino acid sequence identity with outgroup sequences within the same angiosperm order^15^. At this level of divergence, different alignment methods can produce different estimates of evolutionary rates, none of which is obviously superior to the others. For this investigation we employed a multifaceted, *in silico* approach to study the evolution of divergent *rpoA* sequences in three unrelated lineages.

For the Annonaceae and *Passiflora*, both the CDD predictions and *dN*/*dS* values for *rpoA* strongly suggest that the divergent genes are functional. Members of both lineages for which plastome sequences are available and which have highly divergent *rpoA* sequences show evidence of substantial and repeated expansions and contractions of the inverted repeat (IR), including genomic rearrangement in the vicinity of *rpoA*. Illegitimate recombination is a logical cause of the divergence of *rpoA* in *Passiflora* and *Annona*. For Annonaceae, more plastomes (e.g. *Asimina* and *Cananga*) will be needed to determine whether divergence of *rpoA* is consistently associated with large shifts in the IR boundaries.

The *Berberis bealei* plastome shows a similar pattern with a 12 kb expansion of the IR that duplicates 15 genes, including the region where *rpoA* resides^26^. This expansion was noted previously in 26 species of *Berberis* using comparative restriction site and gene mapping^27^. Although Ma *et al.*^26^ reported that *rpoA* was absent from the *B. bealei* plastome, given the similaries between it and the species studied here, we searched for a divergent *rpoA* that could have been overlooked in the original analyses. Indeed, we identified a copy (coordinates 78645–79644, NC_022457) of *rpoA* with 67% nucleotide sequence identity to another member of the same family, *Nandina domestica,* which retained all three functional domains according to a CDD search.

Shifts in IR boundaries in *Pelargonium* have been even more extreme^28^. In *Pelargonium*, *dN*/*dS* values for *rpoA* indicated that this gene is under purifying selection and therefore likely functional. Furthermore the persistence of PEP promoters and the identification of all six PEP sigma factor sequences, but no *rpoA* homolog in the nuclear transcriptome of *Pelargonium x hortorum*^20^ corroborate functionality. The CDD results are less definitive, with all three functional domains predicted for most but not all species. This complex pattern of functional domain conservation is inconsistent with a single loss of *rpoA* function in *Pelargonium*. If indeed failure to predict all three functional domains indicates a lack of function, then multiple independent losses of *rpoA* would be required to achieve the pattern represented in Table 2. In addition to being unparsimonious, this scenario does nothing to explain how *rpoA* may have retained functionality in some clades despite an unparalleled degree of divergence from the outgroup *Eucalyptus*.

In the species with mulitple paralogs, represented by *P. x hortorum*, the IR region has expanded to three times the normal angiosperm size (75,741 bp)^15^. It is possible that once fixed inside the IR these peculiar *rpoA* paralogs become more difficult to purge from the plastome, as the rate of sequence evolution in the IRs is slower than in single copy regions^29^.

*Passiflora biflora*, *Annona cherimola*, *Berberis bealei* and especially Geraniaceae display myriad plastome abnormalities including structural rearrangement, loss of genes and introns, and the divergence of genes that are conserved in almost all other photosynthetic angiosperms^6^,^7^. Illegitimate recombination during plastid DNA repair explains the seemingly opposite nature of the genomic divergence between Geraniaceae genera. For example, in *Erodium* illegitimate recombination led to the deletion of one copy of the IR^30^, whereas in *Pelargonium* it led to an expansion and rearrangement of the IR^15^. In both cases, illegitimate repair of plastid DNA may have caused structural changes that did not delete any genes or their regulatory elements and thus the mutant plastomes were able to reach fixation.

In view of the high levels of sequence divergence of *rpoA* in these four unrelated lineages of angiosperms and the much lower levels of divergence in related species, the question as to why this gene has diverged so significantly remains. We propose that the divergence is a result of two factors, the inherently labile nature of the gene product, which is known from bacteria to be the least conserved of the polymerase subunits^23^, and the high degree of genomic rearrangement by illegitimate recombination in the rearranged plastomes. The *dN*/*dS* values <1 for the Annonaceae, *Passiflora* and *Pelargonium* species included in these analyses also suggest that the divergence of these genes has resulted from a neutral process and is not the result of positive selection. Unlike another gene found to be divergent or missing in Geraniaceae, *accD*, the conserved domains of *rpoA* consist of amino acids dispersed across the ORF rather than a single block of contiguous, conserved amino acids that form the catalytic domain of *accD*. The dispersed nature of the functional domains in *rpoA* may permit substantial divergence of much of the gene, as long as a number of individual non-contiguous, conserved amino acids are undisturbed.

The especially high level divergence in *Pelargonium* clade C2 *rpoA* (Table 2) may be due to gene conversion among paralogs, which is simply a special case of illegitimate recombination. The frequency of gene conversion events is difficult to estimate, but it is sufficiently frequent in section *Ciconium rpoA* sequences to cause the genes to group together by species, rather than by gene, in a phylogenetic reconstruction. The effect of gene conversion overrides the phylogenetic signal one would expect if these genes were evolving independently. The presence of multiple shared pseudogenes of *petD* and *rps11* upstream from the ORFs (Fig. S6) suggests that gene conversion has taken place not only in coding sequences but in intergenic regions as well. We propose that the same error-prone recombination-based DNA repair mechanism likely underlies the divergence of *rpoA* in all four lineages examined, and that this mechanism is likely also responsible for the abnormal fluidity of the IR boundary in *Annona*, *Berberis*, *Passiflora* and *Pelargonium*.

Previous studies have hypothesized that aberrant DNA repair was responsible for accelerated rates of nucleotide substitution, gene and intron loss, and genomic rearrangement of plastid genomes in Geraniaceae^28^,^30^,^31^ and Campanulaceae^32^. With our present findings we propose a more specific hypothesis: These unusual phenomena, including the divergence of *rpoA* and movement of the IR boundaries, are likely due to the failure to suppress illegitimate recombination during replication or repair of plastid DNA, both of which are dependent on recombination^33^.

The Whirly genes encode single stranded DNA binding proteins that suppress illegitimate recombination in *Arabidopsis* and maize^34^. We envision a scenario in which these or other proteins that normally suppress illegitimate recombination in plastids are either insufficiently expressed or compromised in their function. As a result of increased illegitimate recombination, the repeat content of affected plastomes increases, which in turn provides an increasing number of substrates for further illegitimate recombination. The process is brought to an end by increased expression or the spread of alleles that more effectively suppress illegitimate recombination.

As long as illegitimate recombination occurs, nothing precludes it occurring within protein-coding genes and affecting their evolution. As with point mutations, most illegitimate recombination events within protein-coding genes are likely to be deleterious and are subject to purifying selection. However, in the less constrained subset of protein-coding genes that includes *rpoA*, the outcomes of some of these events are more likely to be neutral and arrive at fixation. The unparalleled divergence of the *rpoA* genes in the four lineages discussed here suggests that they evolved not simply through an accumulation of single nucleotide substitutions but also through at least one mechanism capable of causing multiple coincident substitutions and indels. Short homology-dependent illegitimate recombination, as seen in Whirly mutants, induces these types of mutations^34^.

## Material and Methods

### Taxon sampling

Taxon sampling included representatives of the Annonaceae, Geraniaceae, Passifloraceae and associated outgroups (Table 3). For some species of Geraniaceae plastomes have already been completed and published^15^,^21^,^28^,^31^,^35^ and gene sequences were extracted from Genbank. For other Geraniaceae and for Passifloraceae, genes were extracted from draft plastomes and individual gene sequences have been submitted to GenBank (Table 3).

### DNA isolation

Total genomic DNA used for all newly generated sequences was extracted by a modified version (including the use of 2% PVP in the extraction buffer) of the hexadecyltrimethylammonium bromide protocol from Doyle & Doyle^36^.

### Plastome sequencing, assembly and annotation

Sequencing of *Passiflora cirrhiflora* (454), *P. quadrangularis* and *P. biflora* (Sanger) was carried out using products of rolling circle amplification of purified plastomes as described in Jansen *et al.*^37^. Sanger sequence reads were assembled using consed^38^ and 454 reads utilized Newbler^39^ and MIRA^40^ as described in Chumley *et al.*^15^ and Blazier *et al.*^35^. For *Annona* and Geraniaceae, total genomic DNA was sequenced on the Illumina HiSeq 2000 at the Genome Sequence and Analysis Facility (GSAF) at the University of Texas at Austin. Approximately 60 million 100 bp paired-end reads were generated from a sequencing library with ~750 bp inserts. Subsequent to filtering, raw reads were assembled *de novo* with Velvet v. 1.2.07^41^ using a range of kmer sizes from 71 to 93, with and without scaffolding enabled. Plastid contigs were identified by BLAST searches against a database of angiosperm plastid protein-coding genes using custom Python scripts. Nuclear and mitochondrial contigs containing plastid DNA insertions were excluded using 1000x coverage cutoff. Assembly and filtering were performed on the Lonestar Linux Cluster at the Texas Advanced Computing Center (TACC). For all genomes, initial annotation was performed with Dogma^42^ and annotations were checked by comparisons to other annotated plastid genes in Genbank using Geneious 7.0.4 (www.biomatters.com).

### Reverse transcription PCR

Total RNA isolated from *P.* *x* *hortorum* was used for RT-PCR to detect transcription of the *rpoA* ORFs. Newly emergent leaves of *Pelargonium* x *hortorum* cv ‘Ringo White’ were collected from live plants grown in the University of Texas at Austin (UT) greenhouse and frozen in liquid nitrogen. Total RNA was isolated by the same protocol used in Zhang *et al.*^20^. Approximately 1 μg of *P.* *x* *hortorum* DNase-free RNA was thawed on ice and used as the template for reverse transcription PCR (RT-PCR). The RT reactions utilized ImProm-II™ Reverse Transcriptase (Promega, Madison WI) following the manufacturer’s protocol. For each reaction a control reaction was performed where no enzyme was added. *rpoA* mRNA sequences were reverse transcribed from within the *rpoA* ORFs. Products were amplified from the RT template with the forward primers located in the upstream genes, *petD* and *rps11* (Fig. S2). Reverse transcription products, 3 μL each, were used as templates for PCR reactions using the Phusion High-Fidelity DNA Polymerase (Thermo Scientific, Pittsburgh PA) according to the manufacturer’s protocol and MgCl_2_-free buffer. Magnesium chloride concentration was adjusted to 2 mM. Primers were designed manually to amplify transcripts of the two largest *rpoA*-like ORFs in *P. x hortorum*. All primer sequences were selected by visual inspection of the *P. hortorum* plastome sequence and are given in Table S1. Amplification products were Sanger sequenced at the Institute of Cellular and Molecular Biology core facility at the University of Texas at Austin.

### Sequence alignment and rates analyses

Gene sequences were extracted from draft or complete plastomes using the default settings for plastid genes in DOGMA^42^, for *rpoA* sequences, the identity setting was lowered to 25%. All sequence editing and alignment was conducted in Geneious 7.0.4 (www.biomatters.com). Alignment of *rpo* genes was conducted using the L-INS-i algorithm in MAFFT as implemented in Geneious, as a single locally alignable block flanked by long terminal gaps was expected^43^. For other plastid genes, the MAFFT G-INS-i algorithm was used, as a global alignment without large terminal gaps was expected. Individual gene trees were constructed by the same methods as the seven-gene constraint trees described below.

Constraint trees for the three datasets (Annonaceae, Geraniaceae and *Passiflora*) were created using a concatenated nucleotide alignment of seven plastid genes (*rpoA*, *rpoB*, *rpoC1*, *rpoC2*, *ndhF*, *matK* and *rbcL*). For Geraniaceae, Clade C2 species were omitted due to the presence of multiple *rpoA* paralogs. Constraint trees were generated by Garli^44^ using the GTR model in Geneious. Codon alignments were created using MAFFT in Geneious. For the *Pelargonium* data set, three additional alignment algorithms (CLUSTALW, MUSCLE and the Geneious aligner) were used in order to control for alignment error with difficult sequences^45^,^46^. All *dN*/*dS* ratios were calculated using the lineage specific seven-gene constraint tree.

Plastid genes were analyzed with codon-based models to quantify the rates of synonymous (*dS*) and nonsynonymous (*dN*) substitution. Analyses were conducted in PAML^47^ 4.7 on the Lonestar Linux Cluster at TACC using custom Python scripts. Codon frequencies were calculated by the F3×4 model, and a free-ratio model was used to compute *dN*/*dS* values. Transition/transversion and *dN*/*dS* ratios were estimated with the initial values of 2 and 0.4, respectively^48^,^49^. A *dN*/*dS* ratio of 50 was selected as an arbitrary cutoff over which a value was assumed to be an artifact.

### Promoter analysis

The upstream regions of *psbA* and *rbcL* were aligned by MAFFT in Geneious and conserved PEP promoter elements were annotated in accordance with Gruissem and Zurawski^50^. Upstream regions of *Cuscuta obtusiflora*, a parasitic plant lacking PEP, were included for comparison.

### Conserved domain prediction

Conserved domains in *rpoA*-like ORFs were predicted by the Conserved Domain Database at NCBI (CDD v.3.10) at an E-value of 0.01 and low-complexity filters applied^51^.

### Detection of gene conversion

Gene conversion among *Pelargonium rpoA*-like ORFs was investigated both manually and using the ORGCONV algorithm^22^. For manual detection, the alignment was inspected for SNPs shared by two or three *rpoA* paralogs in a single species that were not shared across paralogs in multiple species.

## Additional Information

**How to cite this article**: Blazier, J. C. *et al.* Divergence of RNA polymerase α subunits in angiosperm plastid genomes is mediated by genomic rearrangement. *Sci. Rep.* **6**, 24595; doi: 10.1038/srep24595 (2016).

## Supplementary Material

Supplementary Information

## Figures and Tables

**Table 1:** Summary of conserved domain database (CDD) search results for Annonaceae and Passifloraceae data sets.

Annonaceae Comparison	N-terminal	dimer	β	β′	nt identity (%)	aa identity (%)	ORF length (bp)
***Annona***	Y	Y	Y	Y	57.0	40.7	1,035
*Asimina*	Y	Y	Y	Y	74.5	63.8	1,020
*Calycanthus*	Y	Y	Y	Y	89.0	86.6	1,020
*Cananga*	Y	Y	Y	Y	55.9	38.5	1,143
*Chloranthus*	Y	Y	Y	Y	100.0	100.0	1,002
*Drimys*	Y	Y	Y	Y	90.2	85.4	1,017
*Liriodendron*	Y	Y	Y	Y	91.3	89.8	1,014
*Magnolia*	Y	Y	Y	Y	91.8	89.4	1,014
*Piper*	Y	Y	Y	Y	85.7	80.2	1,017
**Passifloraceae Comparison**					**nt identity**	**aa identity**	**ORF length**
*Hevea*	Y	Y	Y	Y	92.0	88.9	1,023
*Jatropha*	Y	Y	Y	Y	91.8	87.9	1,017
*Linum*	Y	Y	Y	Y	86.9	80.8	1,011
*Manihot*	Y	Y	Y	Y	91.9	87.8	1,029
*Oxalis*	Y	Y	Y	Y	89.8	85.3	1,017
*P. biflora*	Y	Y	Y	Y	53.6	37.4	1,071
*P. ciliata*	Y	Y	Y	Y	93.4	88.8	1,005
*P. cirrhiflora*	Y	Y	Y	Y	93.8	90.6	1,017
*P.quadrangularis*	Y	Y	Y	Y	93.3	89.1	1,017
*Populus*	Y	Y	Y	Y	100.0	100.0	1,017
*Ricinus*	Y	Y	Y	Y	91.9	88.0	996
*Turnera*	Y	Y	Y	Y	80.7	71.0	891

Predictions of the PEP α subunit N-terminus, homodimer interface, beta and beta prime interfaces are indicated (Y = Yes, N = No). The pairwise identity of each sequence with the outgroups *Populus* or *Chloranthus* is given for nucleotide (nt) and amino acid (aa) alignments. Generic names in Annonaceae comparison are in bold; other genera represent related families of magnoliids or the outgroup *Chloranthus*. P. = *Passiflora*; species in bold in Passifloraceae comparison are members of the genus *Passiflora*; other genera are related familes of rosids.

**Table 2:** Summary of conserved domain database (CDD) search results for *Pelargonium* data set.

Outgroup and Geraniales	N-terminal	dimer	β	β′	nt identity (%)	aa identity (%)	ORF length (bp)
*Eucalyptus*	**Y**	**Y**	**Y**	**Y**	100.0	100.0	1,014
*Francoa*	**Y**	**Y**	**Y**	**Y**	92.2	85.8	1,020
*Melianthus*	**Y**	**Y**	**Y**	**Y**	91.3	84.4	1,020
*Viviana*	**Y**	**Y**	**Y**	**Y**	84.0	73.2	1,014
*Hypseocharis*	**Y**	**Y**	**Y**	**Y**	77.8	65.4	1,089
**Pelargonium Clade A1**							
* P. citronellum*	**Y**	**Y**	**N**	**N**	46.0	31.6	885
* P. cucullatum*	**Y**	**Y**	**N**	**N**	46.0	31.6	885
* P. nanum*	**Y**	**Y**	**Y**	**Y**	46.2	31.9	885
* P. quercifolium*	**Y**	**Y**	**Y**	**Y**	46.0	31.6	885
**Clade A2**							
* P. alternans*	**Y**	**Y**	**Y**	**Y**	46.3	31.6	885
* P. echinatum*	**Y**	**Y**	**Y**	**Y**	45.1	32.0	858
* P. fulgidum*	**Y**	**Y**	**Y**	**Y**	44.9	31.4	855
* P. incrassatum*	**Y**	**Y**	**Y**	**Y**	46.2	32.2	885
* P. luridum*	**Y**	**Y**	**Y**	**Y**	46.2	31.6	885
**Clade B**							
* P. australe*	**Y**	**Y**	**Y**	**Y**	44.0	34.1	828
* P. cotyledonis*	**Y**	**Y**	**Y**	**Y**	48.8	33.8	945
* P. exstipulatum*	**Y**	**Y**	**Y**	**Y**	46.0	33.0	879
* P. grossularioides*	**Y**	**Y**	**Y**	**Y**	45.1	31.0	864
* P. reniforme*	**Y**	**Y**	**Y**	**Y**	46.2	32.7	885
**Clade C1**							
* P. dolomiticum*	**Y**	**Y**	**N**	**N**	34.1	24.4	750
* P. trifidum*	**Y**	**Y**	**N**	**N**	32.6	23.5	708
* P. myrrhifolium*	**Y**	**Y**	**N**	**N**	35.1	25.2	714
* P. tetragonum*	**Y**	**Y**	**Y**	**Y**	46.0	29.8	912
* P. worcesterae*	**Y**	**Y**	**Y**	**Y**	46.2	30.1	912
**Clade C2**							
* P. endlicherrianum_578*	**Y**	**Y**	**N**	**N**	32.3	25.0	1,701
* P. endlicherrianum_597*	**Y**	**Y**	**N**	**N**	31.6	25.1	1,737
* P. spinosum_578*	**Y**	**Y**	**N**	**N**	30.1	19.9	1,773
* P. spinosum_597*	**Y**	**Y**	**N**	**N**	35.0	23.0	1,788
* P. transvaalense_597-1*	**Y**	**Y**	**N**	**N**	35.3	21.1	1,788
* P. transvaalense_597-2*	**Y**	**Y**	**Y**	**Y**	36.0	20.5	1,788
* P. transvaalense_597-3*	**Y**	**Y**	**N**	**N**	35.4	21.1	1,788
* P. transvaalense_597-4*	**Y**	**Y**	**N**	**N**	35.5	21.8	1,782
* P. transvaalense_597-5*	**Y**	**Y**	**N**	**N**	35.5	21.4	1,866
* P. transvaalense_597-6*	**Y**	**Y**	**N**	**N**	33.7	19.3	1,788
**Clade C2, sect. Ciconium**							
* P. alchemilloides_521*	**Y**	**Y**	**N**	**N**	34.8	33.5	702
* P. alchemilloides_578*	**Y**	**Y**	**Y**	**Y**	31.8	15.9	1,737
* P. alchemilloides_597*	**Y**	**Y**	**N**	**N**	30.7	14.8	1,794
* P. quinquelobatum_521*	**Y**	**Y**	**N**	**N**	33.6	20.6	1,560
* P. quinquelobatum_578*	**Y**	**Y**	**Y**	**Y**	32.0	15.9	1,737
* P. quinquelobatum_597*	**Y**	**Y**	**N**	**N**	30.7	15.3	1,794
* P. tongaense_521*	**Y**	**Y**	**Y**	**Y**	33.0	20.6	1,554
* P. tongaense_578*	**Y**	**Y**	**Y**	**Y**	31.9	15.7	1,737
* P. tongaense_597*	**Y**	**Y**	**N**	**N**	30.3	14.9	1,815
* P. xhortorum_521*	**Y**	**Y**	**Y**	**Y**	33.4	21.1	1,566
* P. xhortorum_578*	**Y**	**Y**	**Y**	**Y**	31.9	15.9	1,737
* P. xhortorum_597*	**Y**	**Y**	**N**	**N**	30.6	14.9	1,794

Predictions of the PEP α subunit N-terminus, homodimer interface, beta and beta prime interfaces are indicated (Y = Yes, N = No). The pairwise identity of each sequence with outgroup *Eucalyptus* is given for nucleotide (nt) and amino acid (aa) alignments.

**Table 3:** Taxon sampling by data set.

Magnoliids/Chloranthales	Accession numbers
*Annona cherimola*	KU563738
*Asimina incana*	KU645794, KU645799, KU645804, KU645810, KU645815, KU645820, KU645825
*Calycanthus floridus*	NC_004993
*Cananga odorata*	KU645791, KU645796, KU645801, KU645806, KU645812, KU645817, KU645822
*Chloranthus spicatus*	NC_009598
*Drimys granadensis*	NC_008456
*Liriodendron tulipifera*	NC_008326
*Magnolia kwangsiensis*	NC_015892
*Piper cenocladum*	NC_008457
**Malpighiales**	
* Hevea brasiliensis*	NC_015308
* Jatropha curcas*	NC_012224
* Linum usitatissimum*	KU645792, KU645797, KU645802, KU645808, KU645813, KU645818, KU645823
* Manihot esculenta*	NC_010433
* Oxalis latifolia*	EU002528, KF224983, HM850223, EU002248, GQ998560, GQ998561, GQ998562
* Passiflora biflora*	EU017067, KU645807, EU017069, EU017092, EU017096, EU017121, EU017122
* Passiflora ciliata*	JX661956, JX662765, JX664062, JX662034, JX663490, JX664953, JX662679
* Passiflora cirrhiflora*	KU645790, KU645795, KU645800, KU645805, KU645811, KU645816, KU645821
* Passiflora quadrangularis*	KU645791, KU645796, KU645801, KU645806, KU645812, KU645817, KU645822
* Populus trichocarpa*	NC_009143
* Ricinus communis*	NC_016736
* Turnera ulmifolia*	JX664965, JX664074, JX663502, JX662777, JX662690, JX662046, JX661965
**Myrtales/Geraniales**	
* Eucalyptus globulus*	NC_008115
* Francoa sonchifolia*	NC_021101
* Melianthus villosus*	NC_023256
* Viviana marifolia*	NC_007957
* Hypseocharis bilobata*	NC_023260
**Clade A1**	
* Pelargonium citronellum*	KM527888
* Pelargonium cucullatum*	KM527887
* Pelargonium nanum*	KM527896
* Pelargonium quercifolium*	KM527897
**Clade A2**	
* Pelargonium alternans*	NC_023261
* Pelargonium echinatum*	KM527891
* Pelargonium fulgidum*	KM527893
* Pelargonium incrassatum*	KM527894
* Pelargonium luridum*	KU535486-KU535492
**Clade B**	
* Pelargonium australe*	KM459517
* Pelargonium cotyledonis*	KM459516
* Pelargonium exstipulatum*	KM527892
* Pelargonium grossularioides*	KU535493-KU535499
* Pelargonium reniforme*	KU535500-KU535506
**Clade C1**	
*Pelargonium dolomiticum*	KM527889
*Pelargonium trifidum*	KM527898
*Pelargonium myrrhifolium*	KM527895
*Pelargonium tetragonum*	KM527899
*Pelargonium worcesterae*	KU535507-KU535513
**Clade C2**	
* Pelargonium endlicherrianum*	KU535514-KU535522
* Pelargonium spinosum*	KU535523-KU535530
* Pelargonium transvaalense*	KM527900
**Clade C2, sect. Ciconium**	
* Pelargonium alchemilloides*	KU535531-KU535539
* Pelargonium quinquelobatum*	KU535540-KU535548
* Pelargonium tongaense*	KU535549-KU535557
* Pelargonium x hortorum*	NC_008454

Single accession numbers represent complete plastomes and seven accession numbers are for taxa with individual sequences for each gene.

**Table 4:** Revised naming system and basic statistics for *P. x hortorum* and other *sect. Ciconium rpoA* ORFs.

Old *P. x hortorum* ORF name	New ORF name	Length in bp/aa	*P. tongaense (bp/aa)*	*P. alchemill. (bp/aa)*	*P. quinque. (bp/aa)*	pairwise identity (%)	identical sites (%)
ORF574	ORF597	1794/597	1815/604	1794/597	1794/597	99.40	98.80
ORF365	ORF578	1737/578	1737/578	1737/578	1737/578	99.50	99
ORFs332+221	ORF521	1566/521	1554/517	702/233*	1560/519	92.70	87

^*^P. alchemilloides ORF521 homolog ends after 702 bp but is otherwise in frame through conserved stop codon after 1470 bp/490aa.

**Table 5:** Gene conversion events detected by ORGCONV.

Converted Sequence	Donor	Start	End	P-value (L/N)	P-value (L-N)
P_alchemilloides_ORF597	P_alchemilloides_ORF521	130	713	1.13E-07	5.14E-06
P_quinquelobatum_ORF597	P_quinquelobatum_ORF521	119	713	2.30E-10	1.09E-08
P_tongaense_ORF597	P_tongaense_ORF521	124	713	5.52E-10	2.74E-08
Pxhortorum_ORF578	Pxhortorum_ORF521	223	300	1.31E-03	5.20E-03
Pxhortorum_ORF597	Pxhortorum_ORF521	669	713	1.03E-03	1.77E-02

The donor and acceptor of each putative gene conversion event are given along with the coordinates of the converted region and the p-value of the conversion event.

**Table 6:**
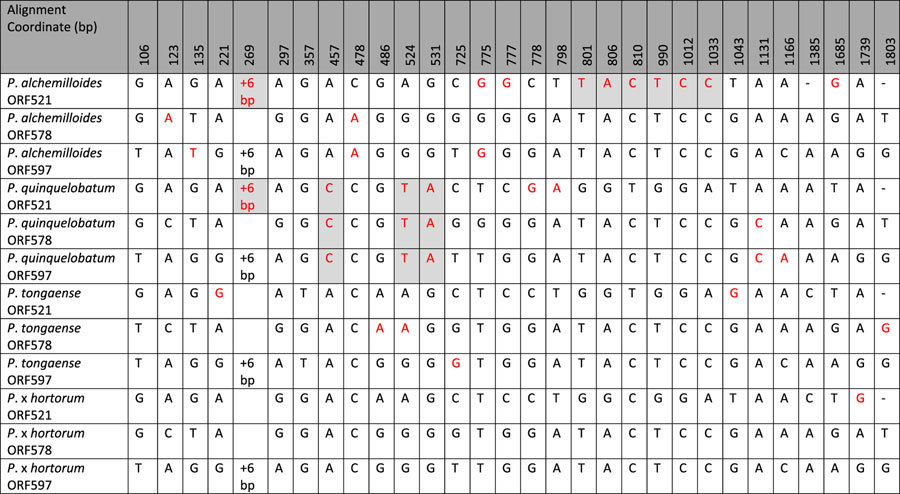
Gene conversion events detected by manual count from an alignment of all 12 ORFs from the four *Pelargonium section Ciconium* species.
