# Supplementary material for: Divergence of RNA polymerase α subunits in angiosperm plastid genomes is mediated by genomic rearrangement
Source: Sci Rep. 2016 Apr 18;6:24595. doi: 10.1038/srep24595 (PMC4834550; doi:10.1038/srep24595)
Supplement: Supplementary Information [file srep24595-s1.pdf]

Supporting Information

Divergence of RNA polymerase α subunits in angiosperm plastid genomes is mediated by genomic rearrangement

J. Chris Blazier, Tracey A. Ruhlman, Mao-Lun Weng, Sumaiyah K. Rehman, Jamal S.M. Sabir, and Robert K. Jansen

The following Supporting Information is available for this article:

Figures S1-S6 and Table S1-S2

Figure S1. Circular map of the plastid genome of *Annona cherimola*. Boxes on the inside and outside of the outer circle represent genes transcribed clockwise and anti-clockwise, respectively. Color coding of genes corresponds to functional units listed in the legend. The inner circle displays the GC content represented by dark gray bars.

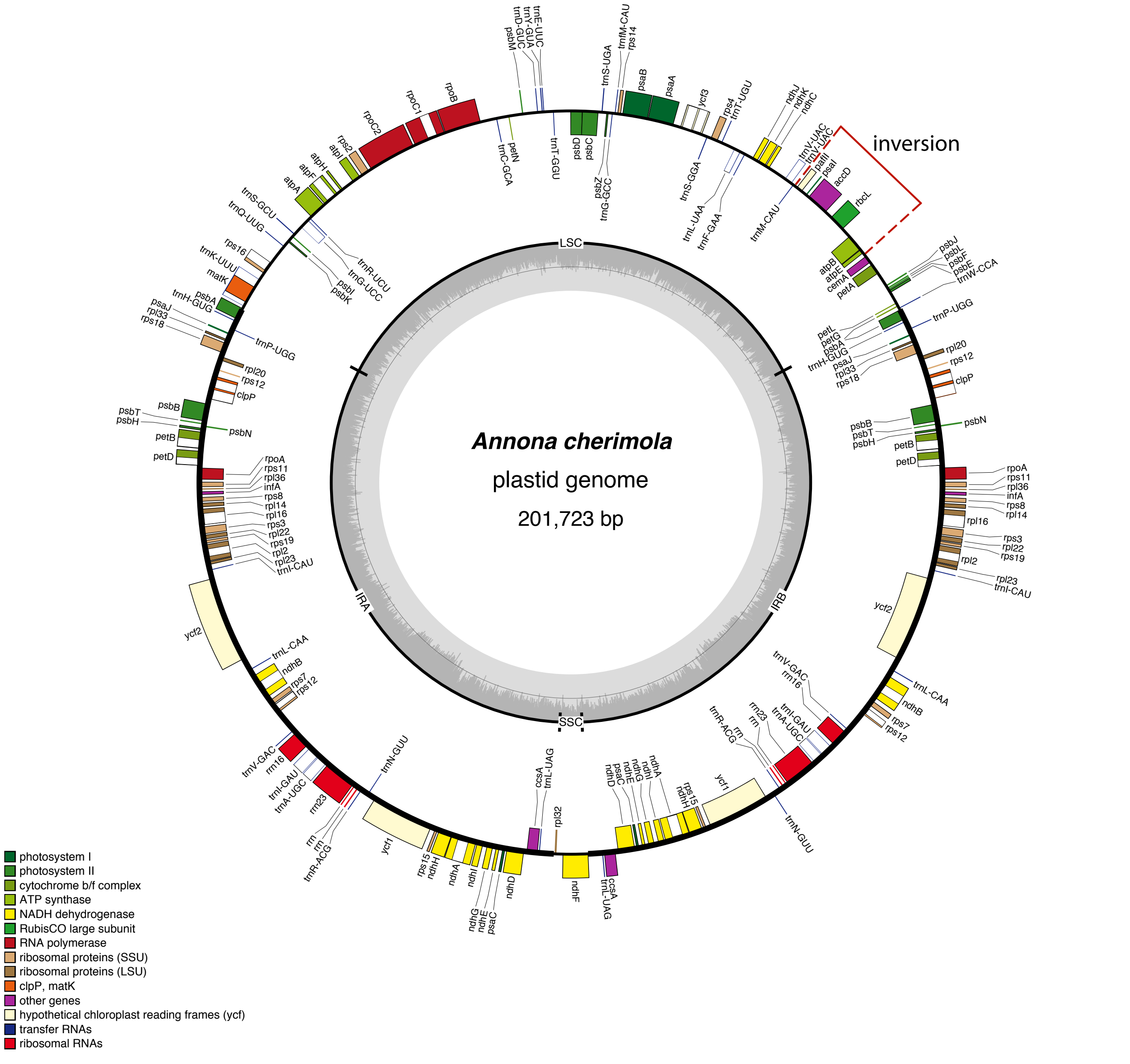

Figure S2. RT-PCR strategy and results for *rpoA* of *Pelargoniun* × *hortorum*. (A) Location of RT-PCR primers for amplification of *P.* × *hortorum* ORF578 and ORF597 transcripts. (B) Agarose gel showing RT-PCR products for P. × *hortorum rpoA* ORFs. Products labeled a and b represent transcripts of ORF578 and ORF597, respectively, while c and d represent polygenic transcripts that include the ORFs and upstream genes *petD* and *rps11*, respectively. Unlabeled products were amplified from the *rbcL* RT template used as a control. The symbols (+) and (-) above each lane indicates the inclusion or absence, respectively, of the RT template in the PCR reaction.

Figure S2

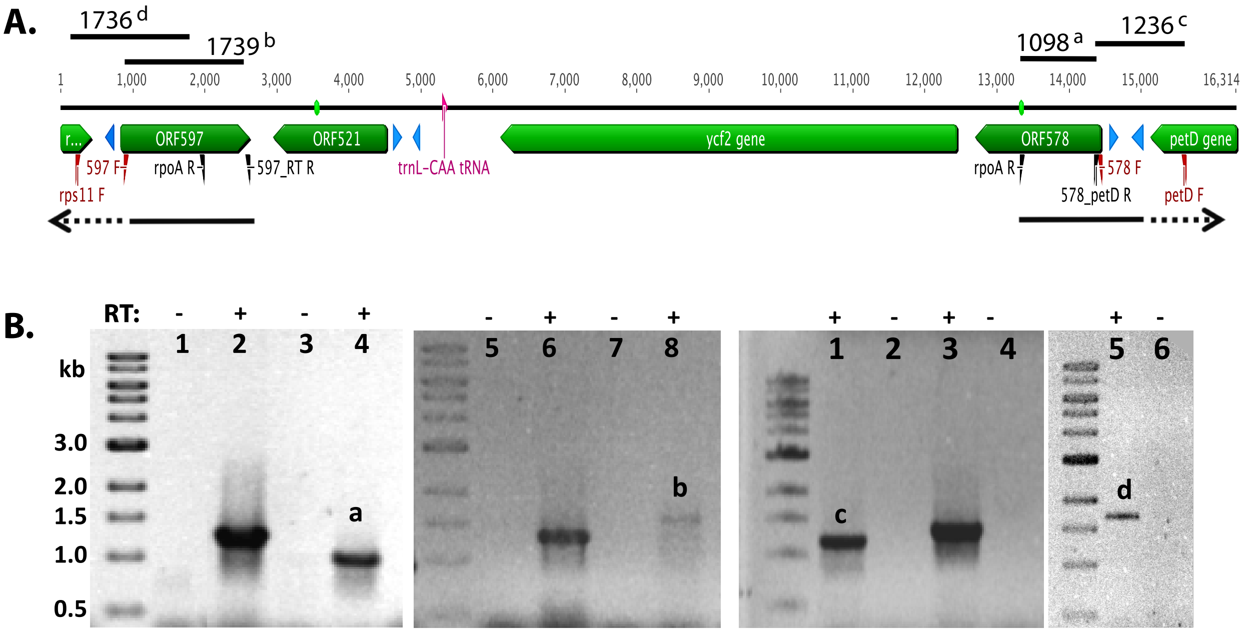

Figure S3. Diagram of the 3' end of the MAFFT alignment of clade B *rpoA* genes. A 6 bp tandem repeat found in all species is shown as dark purple arrows, and a larger 39 bp tandem repeat only present in *P. cotyledonis* also containing the 6 bp repeat is shown in light purple.

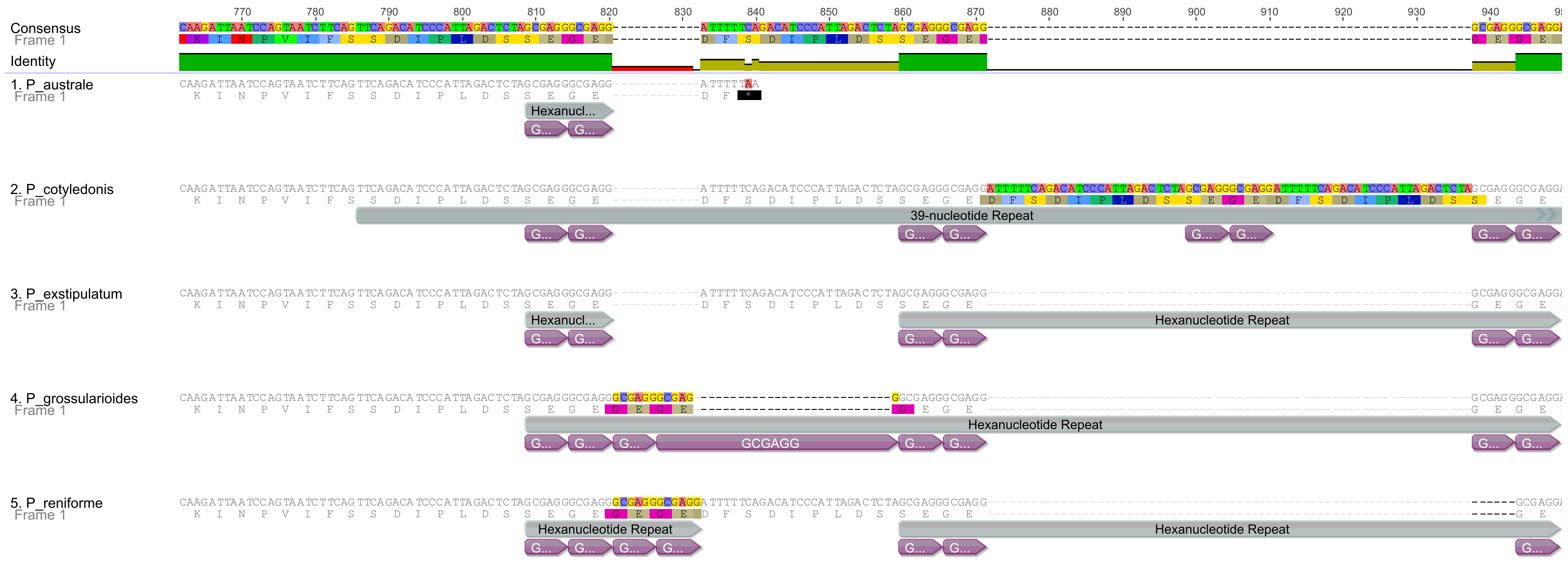

Figure S4. Diagram of the 3' end of the MAFFT alignment of clade A *rpoA* genes. Tandem repeats are shown as purple arrows, and deletions in *P. fulgidum* and *P. echinatum* are indicated by dashes in the coding sequences.

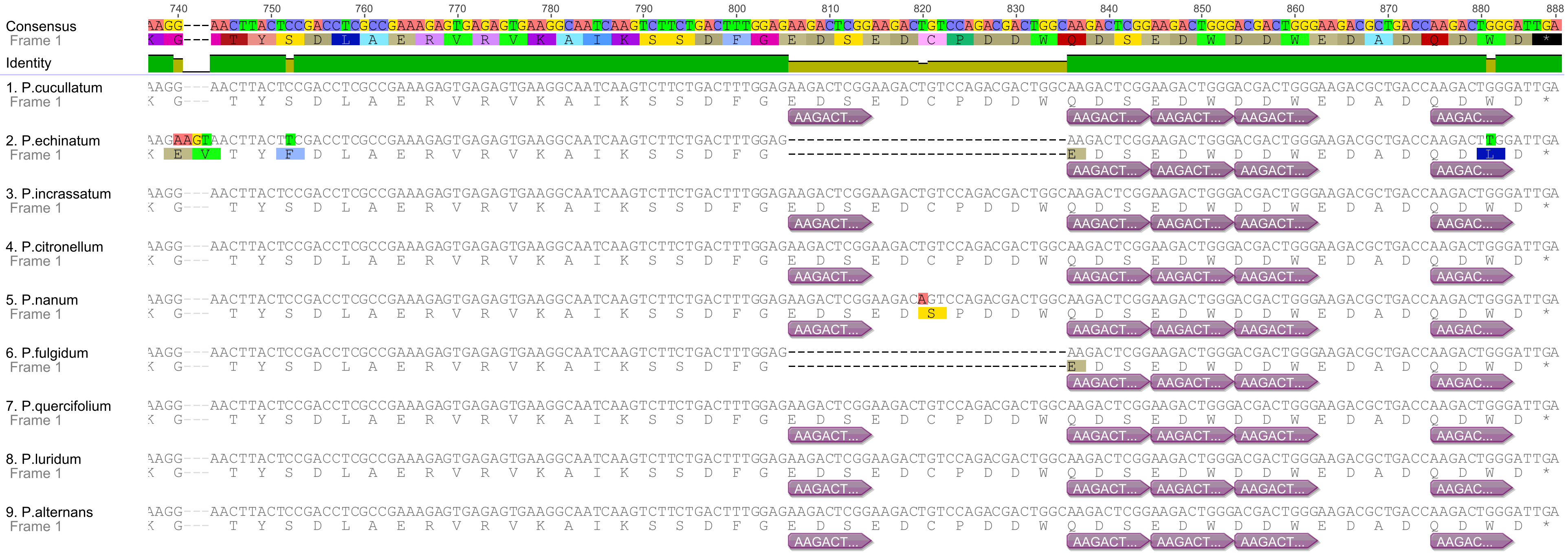

Figure S5. Conserved Domain Database consensus alignment for α subunit interface domains for *Pelargonium × hortorum*, *Annona cherimola* and *Passiflora biflora*. At the top is a diagram representing ORF578 from *P. × hortorum* with subunit interactions domains drawn to scale. Conservation of residues is indicated by grey shading in alignments: 100% similar, black; 80 to 100%, dark grey; 60 to 80%, light grey, less than 60%, no shading. Other species in the alignment are arbitrarily chosen by CDD search to represent diverse bacteria and algae.

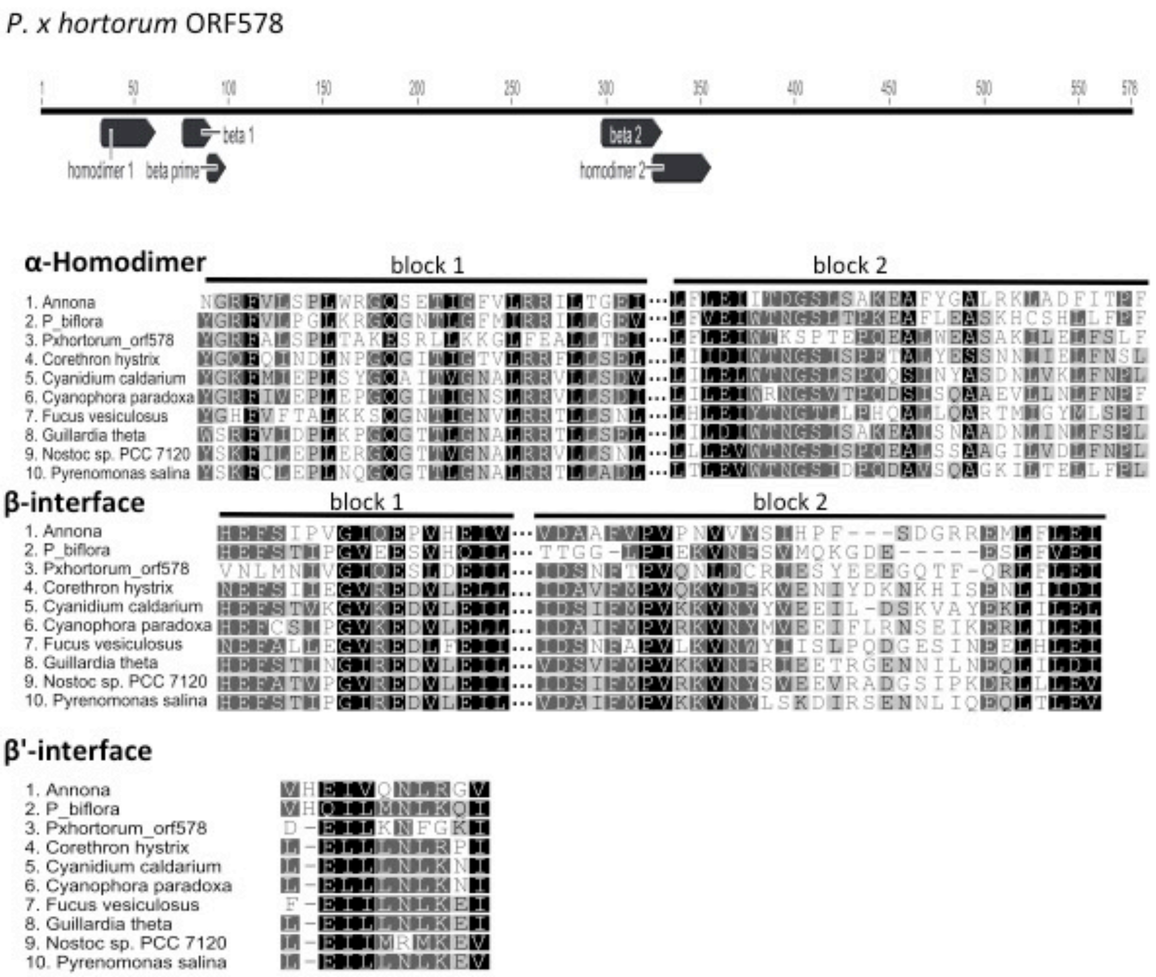

Figure S6. Diagram of the three corrected *rpoA*-like ORFs in the *P.* × *hortorum* plastid genome. The former ORF574 was assigned an upstream ATG start codon (instead of the previously annotated ATT alternative start codon) and renamed ORF597 to reflect the new length in amino acids. ORF221 and ORF332 were joined into a single ORF, ORF521, after a sequencing error was corrected; the insertion of a missing base pair is indicated as a small vertical arrow above the ORF. The former ORF365 had a sequencing error, a missing base pair that caused an apparent frameshift and premature stop codon. After correction the gene is similar in length to the other two ORFs and has been renamed ORF578. The insertion of a missing base pair is indicated as a small vertical arrow above the ORF.

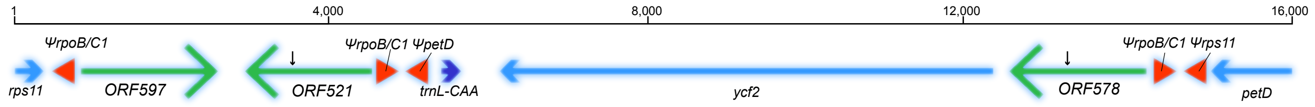

Table S1. RT-PCR oligonucleotide sequences.

Primers for RT-PCR:

Primer for RT reaction: *rpoA*-R: GAATTCTCGATTTCCTCTTTTCCG

Primers for PCR reactions: *petD*-F: CCCGACTTGAATGATCCTG; 578-R: CCACATAGTCCCAGTCT; *rps11*-F: GCTATTCGCACAGTAGTAAC; 597-R: GTCCTTTTCGTTTTC

Table S2. Expanded results supporting Figure 5. Four different algorithms were used in alignment of *Pelargonium* sequences. Values are the ratio of nonsynonymous substitutions to synonymous substitutions (*dN*/*dS*); values > 1 are in bold. Asterisk indicates values > 50.

		Geraniaceae	*Pelargonium*	Clade A/B	Clade A	Clade B	Clade C1
rpoA							
	MAFFT	0.4417	0.2895	0.2717	0.4277	0.4405	0.1558
	MUSCLE	*	0.2241	0.3564	0.3917	0.5216	0.1157
	CLUSTALW	0.5225	0.3317	0.3194	0.4756	0.4073	0.1095
	Geneious	0.4797	0.306	0.2991	0.4364	0.4913	0.1149
rpoB							
	MAFFT	0.3271	0.3397	**1.0804**	**5.9216**	**1.5002**	0.8164
	MUSCLE	0.3153	0.3824	**1.1152**	**5.2133**	**1.3523**	0.813
	CLUSTALW	0.3144	0.385	**1.0178**	**4.7961**	**1.4439**	0.8201
	Geneious	0.3336	0.3451	**1.1462**	**2.5807**	**1.4648**	0.8637
rpoC1							
	MAFFT	0.3249	0.5775	**1.8545**	**2.2963**	**4.1755**	**1.9503**
	MUSCLE	0.3278	0.5671	**1.9809**	**2.53**	**4.0693**	**1.9034**
	CLUSTAL	0.3301	0.5992	**1.9068**	**2.3202**	**2.7216**	**1.8449**
	Geneious	0.3234	0.5772	**1.6394**	**1.6203**	**3.2839**	**1.8638**
rpoC2							
	MAFFT	0.2796	0.3804	**2.1478**	0.9408	0.8104	**1.5229**
	MUSCLE	0.2835	0.3717	**2.1931**	0.9949	0.795	**1.385**
	CLUSTALW	0.2713	0.3887	**2.3449**	0.9751	0.8003	**1.4722**
	Geneious	0.28	0.3679	**2.1464**	**1.1315**	0.7682	**1.271**
matK							
	MAFFT	0.4176	0.3076	0.2874	0.4873	0.4892	0.1306
	MUSCLE	0.4132	0.3116	0.2891	0.4883	0.4905	0.1325
	CLUSTALW	0.4794	0.3489	0.2929	0.4944	0.5338	0.1331
	Geneious	0.453	0.3212	0.2881	0.4864	0.5249	0.1313
ndhF							
	MAFFT	0.1602	0.2563	0.3346	0.2362	0.4186	0.4322
	MUSCLE	0.1598	0.2564	0.3438	0.2312	0.4254	0.4368
	CLUSTAL	0.1706	0.2531	0.3377	0.2362	0.3328	0.4291
	Geneious	0.1402	0.2453	0.339	0.2348	0.4136	0.4715
rbcL							
	MAFFT	0.0304	0.001	0.0634	0.001	0.1309	0.2651
	MUSCLE	0.0304	0.001	0.0634	0.001	0.1309	0.2651
	CLUSTALW	0.0304	0.001	0.0634	0.001	0.1309	0.2651
	Geneious	0.0304	0.001	0.0634	0.001	0.1309	0.2651
